# Investigating the Pretreatment miRNA Expression Patterns of Advanced Hepatocellular Carcinoma Patients in Association with Response to TACE Treatment

**DOI:** 10.1155/2015/649750

**Published:** 2015-02-25

**Authors:** Medhat S. El-Halawany, Heba M. Ismail, Ahmed A. Zeeneldin, Ammar Elfiky, Marwa Tantawy, Mohamed H. Kobaisi, Ikram Hamed, Abdel Hady A. Abdel Wahab

**Affiliations:** ^1^Zoology Department, Faculty of Science, Cairo University, El-Gamaa Street, Giza 12613, Egypt; ^2^Cancer Biology Department, National Cancer Institute, Cairo University, Kasr Al Eini Street, Fom El Khalig, Cairo 11796, Egypt; ^3^Medical Oncology Department, National Cancer Institute, Cairo University, Kasr Al Eini Street, Fom El Khalig, Cairo 11796, Egypt; ^4^Research Department, Children's Cancer Hospital, 1 Seket Al-Emam Street, El-Saida Zeinab, Cairo 11441, Egypt; ^5^Pathology Department, National Institute of Urology and Nephrology, Cairo, Egypt; ^6^Radio-Diagnosis Department, National Cancer Institute, Cairo University, Kasr Al Eini Street, Fom El Khalig, Cairo 11796, Egypt

## Abstract

Hepatocellular carcinoma (HCC) is a lethal malignancy with poor prognosis and limited treatment options. Transarterial chemoembolization (TACE) using chemotherapy agents—doxorubicin and cisplatin—is an accepted treatment option for locally advanced hepatocellular carcinoma. In the current study, we analyzed the expression pattern of a selected panel of 94 miRNAs in archival samples that were collected prior to treatment from 15 Egyptian patients diagnosed with advanced hepatocelleular carcinoma. We observed an overall increase in miRNA expression in HCC samples compared with normal subjects. Out of 94 examined miRNAs, 53 were significantly upregulated while 3 miRNAs were downregulated in HCC samples compared to normal liver samples. Comparing the pretreatment miRNA expression profiles in HCC patients and the patients response to TACE treatment resulted in the identification of a set of 12 miRNAs that are significantly upregulated in nonresponders group. This miRNA panel includes miR-10a-1, miR-23a-1, miR-24, miR-26a, miR-27a, miR-30c, miR-30e, miR-106b, miR-133b, miR-199a, miR-199-3p, and miR-200b. Furthermore, we observed that a panel of 10 miRNAs was significantly associated with patients' survival status at 1 year. These results highlight the potential implications of pretreatment miRNAs expression profiling in prediction of the patients' response to TACE treatment in liver cancer.

## 1. Introduction

Hepatocellular carcinoma (HCC) is the third leading cause of cancer deaths worldwide; its incidence rates vary globally [1]. HCC is a national health concern in Egypt, with rising incidence in the past decade. The annual incidence rate of HCC in Egypt is the highest in Middle Eastern countries ranging from 21.9/100,000 in males to 4.5/100,000 in females according to Population-Based Cancer Registry Data for Middle Eastern Countries [2]. Hepatitis C virus infection is a major aetiology risk factor that predisposes to HCC in Egypt, taking into consideration that Egypt has the highest HCV prevalence in the world with a ratio of about 14.7% among 15–59 years age group [3]. This is along with nonviral aetiology risk factors such as aflatoxin, pesticides, pollution, insulin resistance, steatosis, and to less extent hepatic schistosomiasis [4, 5]. Most of the HCC patients are diagnosed at the intermediate to advanced stages. The difficulty in the treatment of advanced HCC is attributed to pathological heterogeneity and different aetiologies of the disease development. When surgery options are precluded, transarterial chemoembolization (TACE) is one of the options that can be used to treat locally advanced tumours in the liver. In this procedure, an intra-arterial injection of anticancer drugs (usually doxorubicin and/or cisplatin) is administrated immediately prior to embolization [6, 7]. Doxorubicin and cisplatin are commonly used as anticancer drugs, but their clinical efficacy is frequently limited by the development of drug resistance. Molecular markers that can predict response to treatment could help to improve the current chemotherapy regimens. Recent studies described an emerging role of miRNAs in a number of cancers and their involvements in tumour cell response to chemotherapeutic agents (reviewed in [8] and cited references).

miRNAs are posttranscriptional regulators; they govern target genes expression in normal and abnormal cellular activities. miRNAs act predominantly through the degradation of their target RNAs and consequently may affect cellular proteins level in diverse signalling pathways. Many complementary approaches have been adapted to reveal their putative endogenous targets and to assess miRNAs-targets interactions [9]. Deregulated miRNAs are involved in various human diseases including cancer through the regulation of cancer related genes involved in cell cycle progression, apoptosis, and tumour angiogenesis [10].

Multiple miRNAs, participating in common altered signalling pathways, are often deregulated between different types of cancers [11, 12]. Cancer-specific miRNAs are able to classify tumours of different origins. Several studies examined the miRNAs expression profile in different types/stages of human liver cancers and noncancerous specimens [13–15]. Aberrant expression of certain miRNAs has likely contributed to the initiation and progression of hepatocellular carcinoma. In the current study we aimed to investigate the emerging role and predictive value of miRNAs as mediators of treatment response in unresectable HCC Egyptian patients. Patients were treated with TACE using doxorubicin and cisplatin regimen. The expression profiles of 94 miRNAs were analysed in patients archival samples collected prior to treatment. Interestingly, we identified a panel of 12 miRNAs that were significantly deregulated in patients' group of responders compared to nonresponders. Furthermore, we observed an association between 10 miRNAs' expression profile and the patients' survival status at one year. Profiling of these miRNAs in HCC patients prior to treatment may serve as a predictive tool of patients' prognosis including response to treatment and survival; however future studies are required to test this hypothesis.

## 2. Patients and Methods

### 2.1. Patients

The study subjects included 15 patients diagnosed with advanced HCC between 2006 and 2010 at the Egyptian National Cancer Institute. All patients were eligible for TACE treatment according to the institutional standard of care [16]. Patients have received cisplatin (50 mg) and doxorubicin (50 mg) and lipiodol as the immobilizing agent. The current study was approved by the institutional review board and specimens used in the study were obtained prior to treatment under each patient's consent along with approval. A group of 10 healthy subjects (adult living liver donors prior to transplantation) were used as normal controls.

HCC patients basal laboratory values were reasonable at the time of presentation to the study (Table 1). Pain was the presenting symptom in more than 70% of patients. Serum levels of alpha-fetoprotein AFP (cut-off level ≥ 200 ng/mL) were elevated in 46.6% patients. Most patients had a Child-Pugh class of B (73.3%), multiple liver lesions (53%), and grade II histology (68%). Okuda stage II patients' category was the most frequent, 84.6%. Most liver toxicity grades, before starting the treatment, were 1 or 2.

Patients' response to treatment was assessed using the Response Evaluation Criteria in Solid Tumours (RECIST V1.0) [17]. Based on that 5 patients were classified as responders while 10 were classified as nonresponders. The overall survival (OS) of the patients was calculated as the time from diagnosis to death. Progression-free survival (PFS) was calculated as the time from starting TACE treatment until death or last known follow-up. With a median follow-up of 40 months, the median overall survival (OS) and progression-free survival (PFS) of the patients were 15 and 6 months, respectively. Patients' clinicopathological data are detailed in Table 2.

### 2.2. miRNAs Extraction from Paraffin-Embedded Tissues

Paraformaldehyde-fixed, paraffin-embedded (FFPE) tissues obtained from HCC patients (prior to TACE treatment) or from normal controls were used in the study. Total RNA (including miRNAs) from FFPE sections (10 *μ*m/sample) was extracted using miRNeasy FFPE kit as described in the manufacturer's instructions (Qiagen; Hilden, Germany). RNA was purified using RNeasy MinElute spin columns (Qiagen). RNA concentrations were quantified using nanoDrop spectrophotometer (Nanodrop Technologies, Wilmington, DE, USA).

### 2.3. Reverse Transcription and Quantitative Real-Time Polymerase Chain Reaction (qRT-PCR)

Total RNA was reverse-transcribed using miScript RT kit (Qiagen). Reactions were incubated at 37°C for 1 hr followed by inactivation of the reaction by incubation at 95°C for 10 min. For miRNA expression profiling, 1 *μ*L of diluted RT product was used (equivalent to 3 ng) as template in a 10 *μ*L PCR reaction containing 1X SYBR Green master mix (Qiagen), 200 nM miRNA specific forward primer, and 200 nM universal primer. The conditions for qRT-PCR were as follows: 95°C for 10 min, followed by 40 cycles of 95°C for 15 s and 63°C for 30 s. All the RT-qPCR reactions were performed on ViiA 7 real-time PCR system (Applied Biosystems, Foster City, CA, USA). All samples were analysed in duplicate.

### 2.4. Data Analysis

Data were analysed using ΔΔCt comparative method. The threshold cycle (Ct) for each miRNA was used in the analysis. miR-1181-1 was used as an endogenous control and its Ct values was subtracted from the miRNA Ct value to obtain ΔCt. To normalize against normal samples in case of comparing normal and tumour tissues, we calculated [ΔΔCt = ΔCt (tumour) − ΔCt (normal)]. The fold of change was then obtained from the formula: fold of change = 2^−(ΔΔCt)^. Heatmaps and hierarchical clustering were performed with log_2_ fold of change using GENE-E software (Broad Institute, Inc.).

### 2.5. Statistical Analyses

The results were analysed using GraphPad prism computer system (GraphPad software, San Diego, CA, USA). Chi square and Fisher exact tests were used to test the association of miRNA expression with each of the patients' clinicopathological parameters. Statistical analysis comparisons were done with Mann-Whitney or Student's *t*-tests for miRNA expression analysis. A *P* value ≤0.05 was considered significant.

## 3. Results

### 3.1. Profiling of 94 miRNAs Expression Patterns in Egyptian Hepatocellular Carcinoma Patients Compared to Normal Subjects

**Figure 1:**
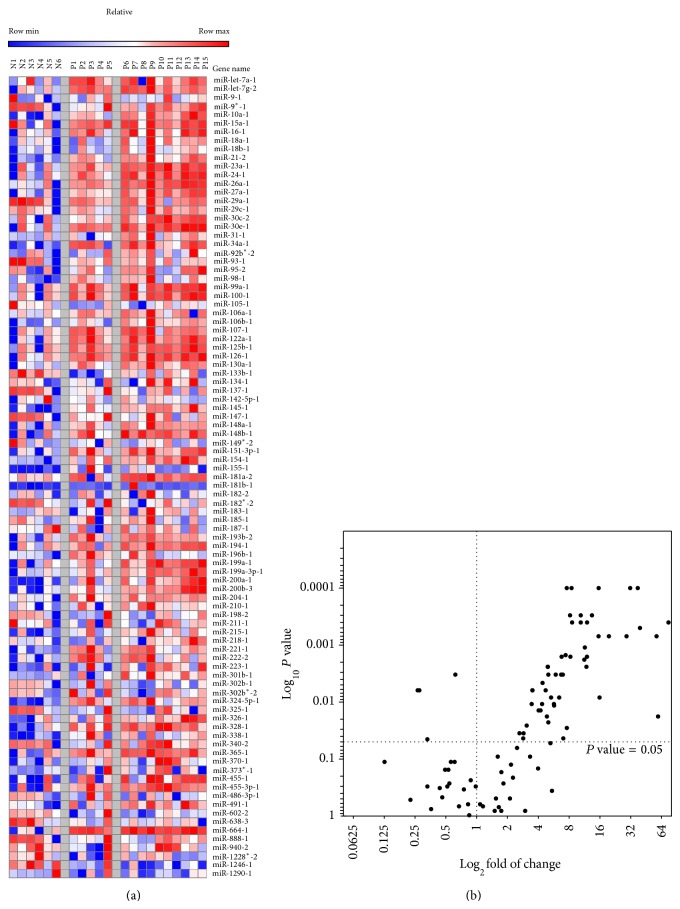
Profiling of 94 miRNA expression patterns in advanced HCC Egyptian patients compared to normal subjects. Expression of 94 miRNAs was determined by real-time PCR in specimens of normal controls (*n* = 10) and HCC patients (*n* = 15). (a) Heatmap of expression profile of 94 miRNAs in HCC patients (P1–P15) and normal donors (N1–N6). (b) Volcano plot of relative gene expression fold of change of the 93 miRNA versus *P* value. Fold of change is represented in log_2_ ratio in the *x*-axis versus *P* value represented in log_10_ ratio in the *y*-axis.

Expression profiles of 94 miRNAs were determined in paraffin tissues obtained from 15 HCC patients prior to TACE treatment with doxorubicin and cisplatin as well as a pool of 10 normal subjects. Of the 94 miRNAs analysed, 53 miRNAs were significantly upregulated (fold change: ≥3; *P* value < 0.05). Only three miRNAs were significantly downregulated (fold change: ≤3; *P* value < 0.05). The calculated values of fold of change in normal and tumour samples are summarized in Supplementary Table  1 (Supplementary Material is available online at http://dx.doi.org/10.1155/2015/649750). The log_2_ fold of change was used to generate expression profile heatmaps using GENE-E software.  Figure 1(a) shows the heatmap for the 93 selected miRNAs.  Figure 1(b) shows Volcano plot of fold of change (represented in log_2_ ratio in the *x*-axis) versus *P* value (represented in log_10_ ratio in the *y*-axis). Volcano plot shows a subset of miRNAs that are differentially deregulated in tumours compared to normal.

**Figure 2:**
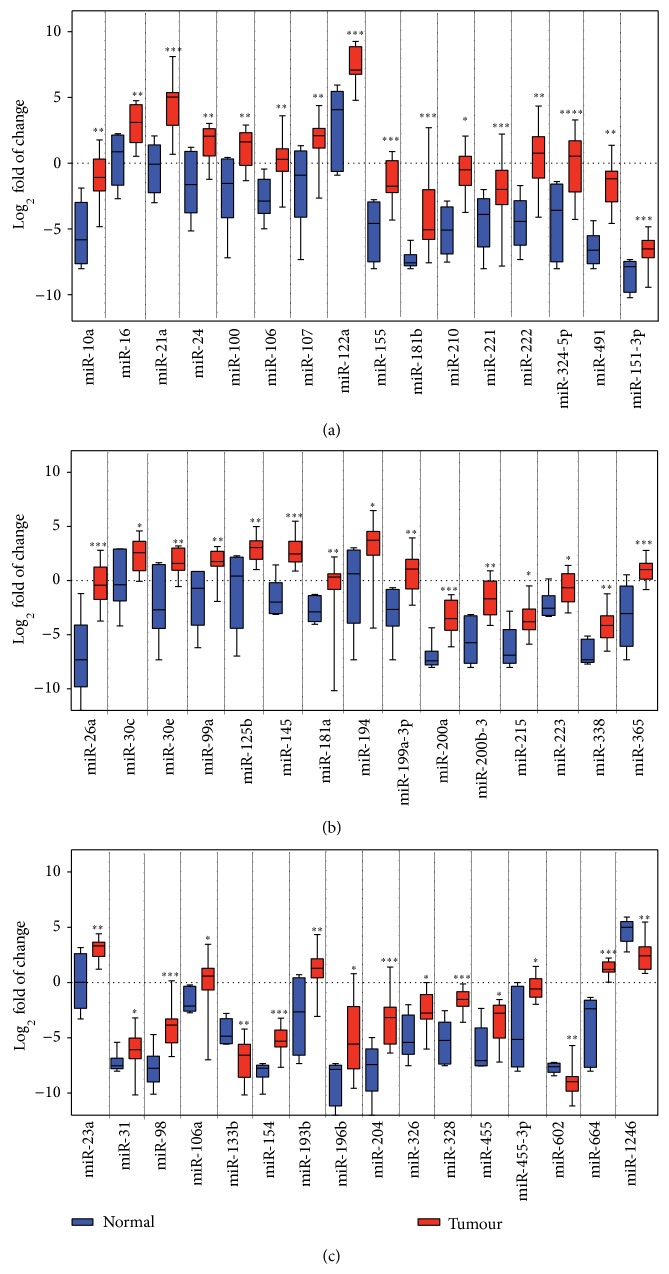
Comparison of miRNA profiles significantly deregulated in our study with published HCC studies. Boxplots of mean miRNA expression after control-based normalization are shown in all panels. Black bars indicate the median. ^*^ *P* < 0.05, ^**^ *P* < 0.01, and ^***^ *P* < 0.001 are based on nonparametric Mann-Whitney tests comparing between miRNA expression levels in normal and tumours. (a) miRNA regulated in our study but not reported in other HCC studies. (b) miRNAs upregulated in our study and upregulated in other HCC studies. (c) miRNAs upregulated in our study and downregulated in other HCC studies.

As far as we know, the current study is the first to profile miRNAs expression pattern in Egyptian HCC patients; we were interested to compare the expression profile of deregulated miRNA in our study with other published reports. In our study, a subset of miRNAs showed expression profiles comparable to the literature including miR-10a, miR-16, miR-21a, miR-24, miR-100, miR-106, miR-107, miR-122a, miR-155, miR-181b, miR-210, miR-221, miR-222, miR-324-5p, miR-491, and miR-151-3p (Figure 2(a)). Another subset of miRNAs was upregulated in our study but was downregulated in other studies including miR-26a, miR-30c, miR-30e, miR-99a, miR-125b, miR-145, miR-181a, miR-194, miR-199a-3p, miR-200a, miR-200b-3, miR-215, miR-223, miR-338, and miR-365 (Figure 2(b)). The expression pattern of the last subset in this group was not previously reported, including miR-23a, miR-31, miR-98, miR-106a, miR-133b, miR-154, miR-193b, miR-196b, miR-204, miR-326, miR-328, miR-455, miR-455-3p, miR-602, miR-664, and miR-1246 (Figure 2(c)).

### 3.2. A Panel of 12 miRNAs Is Significantly Upregulated in TACE Nonresponders Group of HCC Patients

To elucidate if the pretreatment miRNA expression profile could correlate with HCC patients' response to TACE treatment, we compared the normalized pretreatment expression profile of the 94 miRNAs in HCC patients' group of TACE responders to that of nonresponders. The patients with complete response (CR) and partial response (PR) were considered as responders while patients with stable disease (SD) and progressive disease (PD) were categorized as nonresponders of the doxorubicin and cisplatin combined chemotherapy, with a median of 2 cycles per patient. Out of the 15 HCC patients investigated in this study, 5 patients were categorized as responders with response scores of 1-2 while 10 patients were categorized as nonresponders with response scores of 3–5.

**Figure 3:**
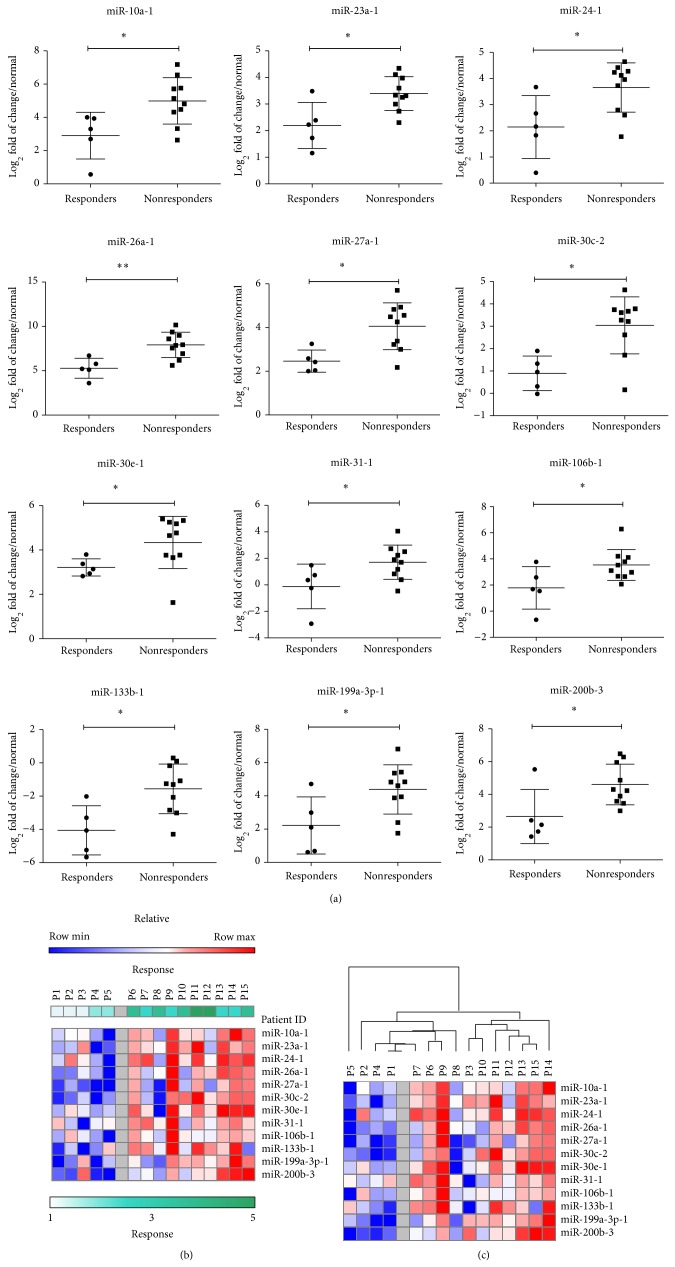
Upregulation of 12 miRNAs panel is significantly associated with patients' poor response to TACE treatment. Expression of miRNAs was determined by real-time PCR in HCC patients. Expression profiles were normalized against normal controls. log_2_ fold of change of miRNA was compared in TACE treatments responders versus nonresponders. (a) Graph shows 12 miRNA that were significantly deregulated in response to treatment. (b) Heatmap of expression profile of 12 miRNAs in TACE responders (*n* = 5) versus TACE nonresponders (*n* = 10). (c) Hierarchical clustering (Pearson correlation, average linkage) of the 12 altered miRNAs in responders and nonresponders groups. Heatmap colors represent relative miRNA expression as indicated in the color key. ^*^ *P* < 0.05 and ^**^ *P* < 0.01 are based on nonparametric Mann-Whitney tests comparing between miRNA expression levels in responders and nonresponders.

Comparing the miRNA expression profiles of responders versus nonresponders group, we identified a panel of 12 miRNAs that were significantly upregulated in the patients group of nonresponders when compared to responders group (Figure 3(a)). This panel includes miR-10a-1 (*P* = 0.0193), miR-23a-1 (*P* = 0.0280), miR-24 (*P* = 0.0280), miR-26a (*P* = 0.0047), miR-27a (*P* = 0.0127), miR-30c (*P* = 0.0127), miR-30e (*P* = 0.0393), miR-106b (*P* = 0.0280), miR-133b (*P* = 0.0193), miR-199a (*P* = 0.0193), miR-199-3p (*P* = 0.0393), and miR-200b (*P* = 0.0280). Heat map of the log_2_ fold of change of the 12 miRNAs in responders versus nonresponders is shown in Figure 3(b). Hierarchical clustering with the 12 altered miRNAs showed a clear separation between responders and nonresponders groups (Figure 3(c)) with exception of patient P8.

### 3.3. miRNA Expression Profile Associated with Patients' Survival Status

**Figure 4:**
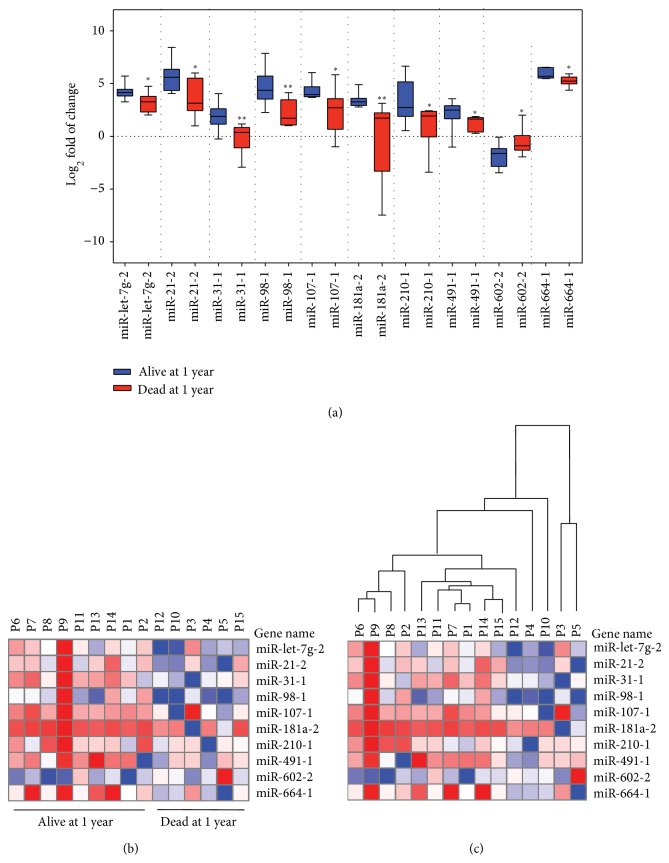
miRNA expression profile associated with patients' survival status at 1 year. Expression of miRNAs was determined by real-time PCR in HCC patients. Expression profiles were normalized against normal controls. Log_2_ fold of change of miRNA was compared in HCC patients at one year of survival. (a) Graph shows 10 miRNAs that were significantly deregulated in association with survival status of patients at one year. (b) Heatmap of expression profile of 10 miRNAs in (a). (c) Hierarchical clustering with the 10 altered miRNAs showed a clear separation between alive and dead groups.

miRNA expressions in HCC cancer samples were compared with patients' clinicopathological parameters including tumour stage and patient's age at diagnosis and survival. Results were tested for statistical significance difference using Chi-square and Mann-Whitney test. No significant association was observed between miRNA expression and patients' age or tumour stage (data not shown). To assess if there is an association between the pretreatment miRNA expression and the patients' survival status, we compared the expression profile of miRNAs and patients' overall survival at one year. A significant association was observed between the patients' survival status at one year and the regulation of 10 miRNAs. This panel included 9 significantly upregulated miRNAs in alive subjects (miR-21-2, miR-3-1-1, miR-98-1, miR-107-1, miR-181a-2, miR-210-1, miR-491-1, and miR-664-1) while one miRNA was significantly downregulated (miR-602-2) (Figure 4(a) and heatmap in Figure 4(b)). Hierarchical clustering using the significantly altered set of 10 miRNAs showed a clear separation between alive and dead subjects (Figure 4(c)).

## 4. Discussion

Approximately 80% of hepatocellular carcinoma cases occur in developing countries with limited resources for health care. HCC is asymptomatic in early stages; thus most of HCC patients present at intermediate to advanced stages. Palliative treatments, including transarterial chemoembolization and systemic chemotherapy, are commonly used in advanced HCC [18]. There are no much information about the potential biomarkers to predict the efficacy and the sensitivity of current treatment regimens in HCC.

The potential roles of miRNAs for affecting drug response in different cancers including HCC were demonstrated and reviewed in many reports. It is not clear how the pretreatment miRNAs expression profile could influence the response to certain chemotherapeutic agents in HCC. To investigate this role, the expression profile of 94 miRNAs from paraffin-embedded tissues was analyzed. In our study, 15 patients with advanced primary HCC were blindly selected prior to local treatment with TACE-doxorubicin and cisplatin combined therapy. No significant differences were observed between the responders and nonresponders groups with regard to clinical and pathological features (data not shown).

53 of the evaluated miRNAs were significantly upregulated. Among the upregulated miRNAs, there is a subset that is consistently overexpressed in HCC (Figure 2(a)) and most likely related to the initiation and development of primary HCC and implicated in cell proliferation, tumour angiogenesis, and invasion. Intriguingly, another subset of the deregulated miRNAs was upregulated in our study but downregulated in other reports, including miR-122, miR-199a-3p, and miR-223 (Figure 2(b)). Another subset of upregulated miRNAs was not reported previously; this includes miR-23a, miR-26, and miR-98 [13–15, 19]. With limited information about the deregulated pattern of miRNAs in advanced HCC, these changes may be a reflection to the change in genes' expression in advanced HCC stage(s) of the analysed samples. Experimental validation is required to confirm this hypothesis/observation.

Most of miRNAs are ubiquitously expressed. miR-122 is abundantly expressed in human adult liver with multiple hepatic functions. As a potential tumour suppressor, miR-122 is frequently downregulated in the most of HCC-derived cell lines and HCC clinical samples. Independent reports also documented a maintained/upregulated expression of miR-122 in HCC with hepatitis viral infections, HBV and HCV, respectively [20, 21]. Our data showed that miR-122 is up to 10-fold higher in all HCC samples compared to normal tissue. There was no complete information regarding the hepatitis viral infection, as primary risk factor, in the enrolled patients of this study, so we cannot passively relate the higher expression of miR-122 to HCV infection or not. Using the available information we tested the correlation between the patients' response to treatment and HCV infection or cirrhosis, but no significant association was obtained (data not shown).

Other tumour suppressor miRNAs, as miR-let7g, miR-199a-3p, and miR-26, were also significantly upregulated in the analysed HCC samples. Notably, miR-199a-5p and miR-199a-3p were shown to be upregulated in the human liver in a fibrosis progression-dependent manner [22]. Upregulation of potential tumour suppressor miRNAs, including miR-122, and miR-199a-3p can be related to liver parameters in the examined advanced HCC patients. This upregulation tendency could be linked to certain cellular mechanism(s) that slow down the cancer progression in certain areas of the liver.

With regard to downregulated miRNAs, our study showed downregulation of 21 miRNAs; only miR-133b, miR-638, and miR-1246 expressions were statistically significant. miR-133 family members (miR-133a, miR-133b) can act as tumour suppressor and are usually underexpressed in tumours [23]. miR-638 and miR-1246 are primate-specific miRNA and expressed during human embryonic development [24]. miR-638 has shown to be down/upregulated in liver cancer tissues [25]. miR-1246 is p53 target and is involved in cancer development [26].

The miRNAs expression patterns and the fold-change values were used to best discriminate between responders and nonresponders. A panel of 12 miRNAs (Figure 3(a)) that were significantly upregulated in the patients group of nonresponders compared to responders was identified. Hierarchical clustering analysis with the 12 altered miRNAs showed a clear separation between responders and nonresponders groups with exception of one responder case (P8). The higher expression and function of the top observed 12 miRNAs might be cooperatively associated with the development of resistance to doxorubicin-cisplatin combined treatment in the present study.

Doxorubicin and cisplatin have wide application for the chemotherapy of various solid tumours. The two drugs can cause DNA damage/adduct and trigger the apoptosis of cancer cells by interfering with different cellular mechanisms [27, 28]. Drug resistance is the major clinical obstacle in the treatment of patients. Acquisition of drug resistance is complex process and may lead to develop cross-resistance to a wide variety of chemotherapeutic drugs, a phenomenon known as multidrug resistance (MDR). It has been shown that the activation of the MDR1 (ABCB1) gene is involved in doxorubicin and cisplatin resistance. Among the identified panel, 3 miRNAs are linked directly to drug resistance in cancer. miR-27a and miR-130b can stimulate MDR1-mediated drug resistance in hepatocellular carcinoma cells [29, 30]. Also, miR-23a can potentiate cells response to drug treatment with altering the level of topoisomerase enzymes [31].

To reveal a common signalling pathway(s) in advanced HCC and the role of posttranscriptional control in drug resistance mechanism validated miRNA targets of the 12-miRNAs were manually curated from relevant published literatures. All the observed 12 miRNAs are shown to be associated with liver fibrosis, cirrhosis and metastasis, and chemotherapeutic drug response (Table 3). Remarkably, the identified miRNAs panel seems to “potentially” interconnect with transforming growth factor-beta (TGF-*β*) induced epithelial-mesenchymal transition (EMT) process in malignant cancers, especially in HCC. Canonical TGF-*β* signalling is a key effector of EMT phenotype in cancer progression and metastasis. However, the relative contribution of other mediators such as p53, PTEN, NF-*κ*B, and Ras signalling is essential to induce EMT phenotype through TGF-*β*. TGF-*β*-mediated EMT plays an important role in the aggressiveness of HCC [32, 33]. Mountain of evidence could show that multistep epithelial-mesenchymal transition process plays a critical role in the development of drug resistance in different types of cancer [34].

miR-10a mediates metastatic properties of HCC by targeting Eph tyrosine kinase receptor, EphA4, thereby regulating EMT process and cell adhesion [35]. TGF-*β* induces the expression of miR-10a/10b in the tissues of brain tumour patients [36]. All three miRNAs of miR-23a~27a~24-2 cluster are among the identified miRNAs panel of this study. The overexpression of miR-23a~27a~24-2 cluster could promote cell growth and attenuate TGF-*β*-induced apoptotic cell death [37]. miR-30 expression was found to be higher in HCC patients with tumour metastasis than in those without [38]. miR-30 inhibits TGF-*β*-mediated EMT in hepatocyte by targeting Snail1 [39]. Overexpression of miR-31 is commonly observed in HCC. It has been reported that miR-31 is a downstream effector of TGF-*β* signalling, and can regulate cancer cell invasion [40]. miR-106b is involved in TGF-*β* signalling pathway; its overexpression is implicated in HCC metastasis through activating EMT process [41]. A recent publication could demonstrate the liver antifibrotic functional role of miR-133a is TGF-*β* dependent [42]. Mammalian target of rapamycin (mTOR) has been identified as miR-199-3p target in HCC. Recent studies could demonstrate that in TGF-*β*-induced EMT, activation of mTOR signalling is required for tumour cell motility and cancer invasion [43].

Members of the miR-200 family were shown to regulate EMT in different cell systems and play important roles in HCC migration by regulating E-cadherin expression [44]. Targeting TGF-*β* signalling pathway is a promising approach in cancer treatment. Different strategies have been developed to interfere with TGF-*β* signalling pathway at different levels. Currently, LY2157299, a novel selective inhibitor of TGF-*β* receptor, is under clinical investigation in advanced HCC patients [45]. As the proper selection of patients for anti-TGF-*β* treatments is critical, the identified miRNA panel can be investigated as potential biomarker for patients selection. Further studies are required to examine the potential impact of the identified panel on patient selection for TGF-*β* treatments.

Additional data analysis could underline a relationship between the pretreatment miRNA expression and patient's survival. We identified another set of 10 miRNAs that includes miR-let-7g-2, miR-21-2, miR-31-1, miR-98-1, miR-107-1, miR-181a-2, miR-210-1, miR-491-1, miR-664-1, and miR-602-2. This 10 miRNAs panel could clearly distinguish alive and dead subjects over one-year period (Figure 4(c)). Unfortunately, we could not have an access to posttreatment tissues to monitor the expression of the observed panel. miR-31 is more likely a common player between the identified drug-resistance-related miRNAs and the 10 miRNAs linked to poor survival in our study.

Overall, we report here the identification of deregulation pattern of miRNA expression in HCC patients compared to normal liver. We initially aimed to link the miRNA expression pattern in patients prior to TACE treatment to with patients' response to treatment. Successfully, we identified a signature of 12 miRNAs that is differentially regulated in nonresponder group of patients. The identified panel could highlight the role of TGF-*β* induced EMT process in HCC progression and development of acquired drug resistance. Furthermore, we report another deregulated set of 10 miRNA in association with patients' poor survival. This attracts a further investigation of reported miRNA panel/signature as a predictor for patients' response to treatment and survival in a larger subset of advanced HCC patients.

## Supplementary Material

Supplementary Table 1: Profiling of 94 miRNA expression in HCC patients compared to normal liver.

## Figures and Tables

**Table 1:** Baseline laboratory values in 15 patients with advanced HCC treated with TACE.

Parameter	Mean ± SD	Median (IQR)
Total bilirubin (mg/dL)	1.2 ± 0.6	0.9 (0.7–1.7)
Albumin (mg/dL)	3.1 ± 0.6	3.1 (2.675–3.575)
AST (U/mL)	53.1 ± 27.5	51.5 (32.8–69.3)
ALT (U/mL)	46.1 ± 22.4	46.0 (30.3–61.0)
INR	1.2 ± 0.3	1.2 (1–1.3)
Creatinine (mg/dL)	0.8 ± 0.2	0.8 (0.7–0.9)
Hemoglobin (g/dL)	13.3 ± 1.9	13.5 (12.5–14.8)
WBC (×1000/mL)	6.3 ± 1.3	6.1 (5.4–7.3)
Platelets (×1000/mL)	174.2 ± 56.2	172 (132–199)

SD: standard deviation, IQR: interquartile range, AST: aspartate transaminase, ALT: alanine transaminase, INR: international normalized ratio, AFP: alpha-fetoprotein, and WBC: white blood cells.

**Table 2:** Clinicopathological characteristics of 15 patients with advanced HCC treated with TACE.

Patient ID	Age	HCV/cirrhosis	Presentation	AFP (ng/mL)	Grade	TNM stage	Child-Pugh class	OKUDA stage	CLIP	Response score
Responders										
P1	49.00	+/+	Jaundice	16.00	2.00	T1	B	2	3	1.00
P2	42.00	ND	Pain	3.00	2.00	T1	B	ND	1	1.00
P3	52.00	ND	Pain	969.00	ND	T3b	B	2	4	1.00
P4	52.00	+/−	ND	107000	3.00	T1	B	2	4	2.00
P5	50.00	−/+	ND	342.00	3.00	T1	B	ND	1	2.00

Nonresponders										
P6	43.00	ND	Pain	276.00	2.00	T2	ND	2	1	4.00
P7	57.00	−/+	ND	4.00	2.00	T1	B	1	1	3.00
P8	59.00	−/+	Pain	ND	2.00	T2	A	2	2	4.00
P9	57.00	−/+	ND	28.00	ND	T2	B	2	2	3.00
P10	52.00	ND	Pain	90.00	1.00	T3a	B	2	3	4.00
P11	61.00	ND	Ascites	350.00	2.00	T2	B	2	2	5.00
P12	58.00	−/+	ND	2580.00	ND	T3a	B	2	4	5.00
P13	67.00	ND	Recurrence	978.00	2.00	T3a	A	2	3	3.00
P14	56.00	+/+	ND	ND	2.00	T1	A	1	0	3.00
P15	58.00	−/+	Pain	10.00	2.00	T2	B	2	3	4.00

TNM: tumor, node, and metastases; AFP: alpha-fetoprotein; ND: not defined; CLIP: Cancer of the Liver Italian Program.

**Table 3:** The literature suggested functions of the 12 miRNAs in HCC.

miRNA	Function
miR-10a	Is involved in metastatic process of HCC.

miR-23a/24/27a	This cluster can promote hepatic metastasis.

miR-26a	Acts as a tumor suppressor in liver; patients with lower miR-26 level have shorter overall survival.

miR-30c/30e	miR-30 family expression is significantly expressed in HCC patients with metastasis.

miR-31	Is significantly expressed in HCC patients and its expression is correlated with liver cirrhosis.

miR-106b	Plays a critical role in development of HCC metastasis.

miR-133b	Plays a role in the development of HCC by regulating CD133(+) liver tumor initiating cells.

miR-199a-3p	Is associated with progression of liver fibrosis and can influence the doxorubicin sensitivity of human hepatocarcinoma cells.

miR-200b	Can mediate hepatocellular carcinoma cell migration, closely associated with progression of liver fibrosis.
