# Supplementary material for: Investigating the Pretreatment miRNA Expression Patterns of Advanced Hepatocellular Carcinoma Patients in Association with Response to TACE Treatment
Source: Biomed Res Int. 2015 Feb 25;2015:649750. doi: 10.1155/2015/649750 (PMC4355598; doi:10.1155/2015/649750)
Supplement: Supplementary file 1 — Supplementary Table 1: Profiling of 94 miRNA expression in HCC patients compared to normal liver. [file 649750.f1.pdf]

Supplementary data

Supplementary table 1: Profiling of 94 miRNA expression in HCC patients compared to normal liver. Shown are mean fold of change for each miRNA normalized against the internal control. Values were compared between normal and tumour samples using Mann- Whitney statistical test.

miRNA	Normal (mean)	Tumour (Mean)	Tumour/Normal	Mann-Whitney(p value)
**miR-let-7a-1**	1.733766117	4.302558838	2.481625863	ns
**miR-let-7g-2**	0.296638009	2.252281983	7.592695183	0.0001
**miR-9-1**	0.161966999	0.070671683	-2.291823153	ns
**miR-9*-1**	0.003552264	0.002933475	-1.210940687	ns
**miR-10a-1**	0.072384589	0.81570989	11.26910988	0.0018
**miR-15a-1**	0.575112776	0.924256083	1.607086682	ns
**miR-16-1**	2.230926661	11.90016725	5.334181289	0.0083
**miR-18a-1**	0.041269105	0.164375891	3.983025389	ns
**miR-18b-1**	0.032962846	0.109724892	3.328744519	ns
**miR-21-2**	1.457822407	46.12126332	31.63709317	0.0001
**miR-23a-1**	2.800671302	9.833495815	3.511120997	0.0062
**miR-24-1**	0.811616019	3.832645082	4.722239327	0.0062
**miR-26a-1**	0.080422063	1.574095195	19.57292722	0.0007
**miR-27a-1**	0.251003154	1.697866489	6.76432332	0.0016
**miR-29a-1**	12.15460972	6.289995043	-1.932371907	ns
**miR-29c-1**	6.290256719	9.715823886	1.544583046	ns
**miR-30c-2**	2.897723006	7.600443391	2.622901974	0.0352
**miR-30e-1**	1.048085005	4.415497424	4.212919183	0.014
**miR-31-1**	0.008212886	0.0234246	2.852176292	0.0433
**mir-34a-1**	0.059472455	3.402621436	57.21340114	0.0007
**miR-92b*-2**	0.012174817	0.021253402	1.745685592	ns
**miR-93-1**	6.756097398	2.230931667	-3.02837487	ns
**miR-95-2**	0.048176754	0.104436842	2.16778495	ns
**miR-98-1**	0.009576793	0.148619057	15.51866622	0.0007
**miR-99a-1**	0.81862938	4.047828313	4.944640897	0.0024
**mir-100-1**	0.570381412	3.381369817	5.928260885	0.0033
**miR-105-1**	0.029104262	0.003669802	0.126091556	ns
**miR-106a-1**	0.405346374	2.034472208	5.019095619	0.0227
**miR-106b-1**	0.250965908	2.069964377	8.247990316	0.0016
**miR-107-1**	0.914187278	6.112901157	6.686705562	0.0033
**miR-122a-1**	23.00685289	239.2203639	10.39778735	0.0003
**miR-125b-1**	2.002917885	9.95558003	4.970538285	0.0024
**miR-126-1**	1.789850257	15.3335566	8.566949407	0.0004
**miR-130a-1**	0.194321319	2.214724311	11.39722763	0.0011
**miR-133b-1**	0.05858371	0.016169437	-3.623113721	0.0062
**miR-134-1**	0.003754056	0.006541087	1.742405089	ns
**miR-137-1**	0.003552264	0.001748351	-2.031779767	ns
**miR-142-5p-1**	0.699091013	0.251259398	-2.782347723	ns
**miR-145-1**	0.690410163	9.250611971	13.3987193	0.0003
**miR-147-1**	0.003556245	0.002670476	-1.331689229	ns
**miR-148a-1**	2.732522406	13.70589859	5.015841245	0.0033
**miR-148b-1**	0.135245384	0.595762517	4.405048802	0.0046
**miR-149*-2**	0.074614025	0.016844254	-4.429642596	ns
**miR-151-3p-1**	0.061855721	0.643196576	10.39833601	0.0004
**miR-154-1**	0.004304995	0.036393681	8.453827452	0.0001
**miR-155-1**	0.007136333	0.53410415	74.84294473	0.0004
**miR-181a-2**	0.198959302	1.274609069	6.406380893	0.0083
**miR181b-1**	0.050262627	33024185.07	657032615.3	0.0001

Supplementary table 1: Continued

miRNA	Normal (mean)	Tumour (Mean)	Tumour/Normal	Mann-Whitney(p value)
**miR-182-2**	0.021711726	0.064718948	2.980829295	ns
**miR-182*-2**	0.003673784	0.002242088	-1.638554614	ns
**miR-183-1**	0.016789092	0.035503858	2.114697959	ns
**miR-185-1**	0.413806049	0.621017893	1.50074629	ns
**miR-187-1**	0.014609207	0.009822902	-1.48725986	ns
**miR-193b-2**	0.540741925	3.762768962	6.958530097	0.0033
**miR-194-1**	3.081290825	17.54003896	5.692432152	0.0108
**miR-196b-1**	0.003555944	0.210373503	59.16107981	0.0179
**miR-199a-1**	0.286174148	1.390881475	4.860262487	0.0179
**miR-199a-3p-1**	0.270987375	3.24307083	11.96760856	0.0016
**miR-200a-1**	0.012574963	0.150663192	11.98120364	0.0004
**miR-200b-3**	0.048065166	0.567700257	11.81105359	0.0024
**miR-204-1**	0.009979174	0.290701023	29.1307707	0.0007
**miR-210-1**	0.093916289	0.714983954	7.612991963	0.0284
**miR-198-2**	0.003563696	0.003491736	-1.020608544	ns
**miR-211-1**	0.019384041	0.016417977	-1.180659573	ns
**miR-215-1**	0.032984158	0.143767789	4.358692035	0.0108
**miR-218-1**	0.012997207	0.070814628	5.448449476	ns
**miR-221-1**	0.090600219	3.572610282	39.43268936	0.0005
**miR-222-2**	0.147849725	2.348173975	15.88216665	0.0083
**miR-223-1**	0.323491384	0.928917157	2.871536003	0.0353
**miR-301b-1**	0.007837431	0.041275823	5.266499017	ns
**miR-302b-1**	0.003552264	0.006489196	1.82677744	ns
**miR-302b*-2**	0.003664613	0.006035627	1.64700239	ns
**miR-324-5p-1**	0.016350843	0.612598674	37.46587647	<0.0001
**miR-325-1**	0.003648086	0.002044912	-1.783981802	ns
**miR-326-1**	0.071401268	0.287875658	4.031800355	0.014
**miR-328-1**	0.050614564	0.413923104	8.177944632	0.0003
**miR-338-1**	0.012235863	0.090565992	7.401684259	0.0015
**miR-340-2**	0.004415594	0.004773253	1.080999106	ns
**miR-365-1**	0.436159526	2.494133214	5.718396751	0.0115
**miR-370-1**	2.77208023	19.46965444	7.023481581	0.0433
**miR-373*-1**	0.0064713	0.014643055	2.262768808	ns
**miR-455-1**	0.043922006	0.151516953	3.449681974	0.0108
**miR-455-3p-1**	0.313360798	0.966219848	3.083410095	0.0258
**miR-486-3p-1**	0.003620291	0.004176428	1.153616644	ns
**miR-491-1**	0.003739279	0.013097869	3.502778915	0.0062
**miR-602-2**	0.004996373	0.003095047	-1.614312636	0.0033
**miR-638-3**	1.45245239	0.479448174	-3.029425218	0.0449
**miR-664-1**	0.174755992	2.720457874	15.56717941	0.0001
**miR-888-1**	0.00355383	0.001882618	-1.887706435	ns
**miR-940-2**	1.223357045	1.07232405	-1.140846412	ns
**miR-1228*-2**	0.010069424	0.005450612	-1.847393113	ns
**miR-1246-1**	31.81663289	8.454801612	-3.763143638	0.0062
**miR-1290-1**	0.020147156	0.009294177	-2.167718049	ns
